# A Compartmental Model to Investigate Local and Global Ca^2+^ Dynamics in Astrocytes

**DOI:** 10.3389/fncom.2018.00094

**Published:** 2018-11-30

**Authors:** Evan Cresswell-Clay, Nathan Crock, Joël Tabak, Gordon Erlebacher

**Affiliations:** ^1^Computational Intelligence Lab, Department of Scientific Computing, Florida State University, Tallahassee, FL, United States; ^2^Institute of Biomedical and Clinical Science, University of Exeter Medical School, Exeter, United Kingdom

**Keywords:** calcium spike, spatiotemporal dynamics, diffusion, IP3 receptor, endoplasmic reticulum, glia

## Abstract

Intracellular Ca^2+^ dynamics in astrocytes can be triggered by neuronal activity and in turn regulate a variety of downstream processes that modulate neuronal function. In this fashion, astrocytic Ca^2+^ signaling is regarded as a processor of neural network activity by means of complex spatial and temporal Ca^2+^ dynamics. Accordingly, a key step is to understand how different patterns of neural activity translate into spatiotemporal dynamics of intracellular Ca^2+^ in astrocytes. Here, we introduce a minimal compartmental model for astrocytes that can qualitatively reproduce essential hierarchical features of spatiotemporal Ca^2+^ dynamics in astrocytes. We find that the rate of neuronal firing determines the rate of Ca^2+^ spikes in single individual processes as well as in the soma of the cell, while correlations of incoming neuronal activity are important in determining the rate of “global” Ca^2+^ spikes that can engulf soma and the majority of processes. Significantly, our model predicts that whether the endoplasmic reticulum is shared between soma and processes or not determines the relationship between the firing rate of somatic Ca^2+^ events and the rate of neural network activity. Together these results provide intuition about how neural activity in combination with inherent cellular properties shapes spatiotemporal astrocytic Ca^2+^ dynamics, and provide experimentally testable predictions.

## 1. Introduction

In the last 25 years, the prevailing notion that astrocytes serve purely as structural and metabolic support has evolved to viewing them as units that interact with neuronal inputs and alter neuronal activity. This new paradigm of astrocyte function emerged after it was discovered that astrocytes possess Ca^2+^ excitability in the form of Ca^2+^ spikes and oscillations. These can be triggered by neuronal activity and the subsequent release of neurotransmitters and increase in extracellular K^+^. Following Ca^2+^ activity, astrocytes can release gliotransmitters that act at the synaptic or perisynaptic level to strengthen or weaken synapses, or to change neuronal activity. This fueled a number of modeling studies that investigated the mechanisms through which synaptic activity triggers Ca^2+^ spikes in astrocytes and how, in turn, the astrocyte response affects the synapses (Nadkarni and Jung, 2007; Postnov et al., 2007; De Pitta et al., 2009; Wade et al., 2011; Tewari and Majumdar, 2012; Khalid et al., 2017). These studies naturally focused on temporal Ca^2+^ dynamics.

Astrocyte-to-neuron signaling directly and indirectly impacts the dynamics of synapses and neurons (Halassa et al., 2009b; Henneberger et al., 2010; Kimelberg and Nedergaard, 2010; Perea and Araque, 2010). These effects can have a short or long duration, and they can affect single or multiple neurons (Halassa et al., 2009a). Paralleling this multiplicity of actions on different temporal and spatial scales, recent *in vivo* imaging of astrocyte Ca^2+^ activity in response to neuronal activity has revealed a spatiotemporal hierarchy of Ca^2+^ events. They range from fast Ca^2+^ fluctuations in the astrocyte periphery to Ca^2+^ spiking across the astrocyte branches and in the cell body (Di Castro et al., 2011; Araque et al., 2014; Kanemaru et al., 2014; Rusakov et al., 2014; Bindocci et al., 2017).

These spatiotemporal events interact in complex ways with governing rules that are important to clarify (Volterra et al., 2014; Bazargani and Attwell, 2016; Bindocci et al., 2017) since they may underlie different classes of astrocyte-to-neuron signaling. Recent work suggests that the most distal processes, which receive synaptic inputs, possess channels that allow fast, localized extracellular Ca^2+^ influx (Di Castro et al., 2011; Bindocci et al., 2017). However these peripheral perisynaptic processes lack the Ca^2+^ stores to trigger Ca^2+^-induced Ca^2+^ release (CICR) (Patrushev et al., 2013; Rusakov, 2015). Thus, wider Ca^2+^ events resulting from CICR are only observed in larger, more proximal processes, away from neuronal synapses (Rusakov, 2015). Bindocci et al. (2017) have used three-dimensional imaging and reconstructions of astrocytes to describe fast and local Ca^2+^ events scattered in the astrocyte gliapil (optically unresolved perisynaptic processes) and the larger events occuring in the astrocyte core (soma and major processes). Of those larger events, a much greater fraction occured in major processes than in the soma. Occasionally, global events that invaded most of the core were observed. These events were not sweeping waves, but were triggered by the simultaneous occurence of Ca^2+^ events in multiple loci, generally in the gliopil, and presumably due to synchronous neuronal activity.

This diversity and complexity of Ca^2+^ events calls for spatial models to understand astrocyte Ca^2+^ dynamics and how they affect neurons and synapses. To date, there are only a handful of spatial models designed to study the spatio-temporal Ca^2+^ dynamics in astrocytes. Kang and Othmer (2009) developed a 2D spatial model with realistic cellular morphology. Their goal, however, was not to study the dynamics of Ca^2+^ events within an astrocyte, but to study how network connectivity, IP_3_ diffusion, and ATP transport affected the propagation of Ca^2+^ waves across astrocyte networks. Wu et al. (2014) modeled Ca^2+^ and IP_3_ dynamics based on a temporal single point model (Ullah et al., 2006) extended to a homogeneous square domain. This model could reproduce the power-law distribution of Ca^2+^ event duration and intensity observed in hippocampal astrocytes, demonstrating that scale-free Ca^2+^ dynamics can arise from intracellular IP_3_ diffusion. Gordleeva et al. (2018) developed a multi-compartmental model of an astrocyte with realistic morphology. They stimulated the most distal compartments on each of their astrocytic processes and considered how the resulting Ca^2+^ signal propagated toward the soma. Synchronous stimulation of multiple processes was necessary for activation of the astrocyte soma, suggesting that astrocytes can serve as detectors of spatial synchronization in a neural network. Finally, Savtchenko et al. (2018) developed a compartmental astrocyte-model builder to reproduce astrocyte morphology and function with high detail. They were able to fit parameters of such a model to physiological data, and demonstrated the use of the model to examine the role of fine details of astrocyte functional morphology, such as the distance between IP_3_ receptor clusters, on Ca^2+^ dynamics.

Here, we develop an astrocyte compartmental model to study how stochastic Ca^2+^ influx due to neuronal activity triggers Ca^2+^ spikes in the main astrocyte processes and how this in turn triggers Ca^2+^ spikes in the soma and global events involving the soma and most major processes. This is similar to the work of Gordleeva et al. (2018), but here we distinguish between fast Ca^2+^ events due to synaptic activity and slower events involving CICR, inspired by the hierarchy of Ca^2+^ events described by Araque et al. (2014). We use a model of complexity intermediate between the single point model of Postnov et al. (2007) and the model of Gordleeva et al. (2018). Keeping the number of compartments and biophysical complexity to a minimum limits the number of variables and free parameters, providing better intuition and understanding of the model dynamics. We use this simple model architecture to ask the following questions:

1. How are neuronal inputs integrated to produce Ca^2+^ spikes in the astrocyte soma?
2. How do neuronal input properties, such as their correlations and distribution over the astrocyte, facilitate Ca^2+^ spikes in the soma and global events involving most processes?
3. How do diffusive properties of the cytosol and Ca^2+^ stores impact the production of soma Ca^2+^ spikes?

## 2. Methods and model description

### 2.1. Compartmental model

**Figure 1:**
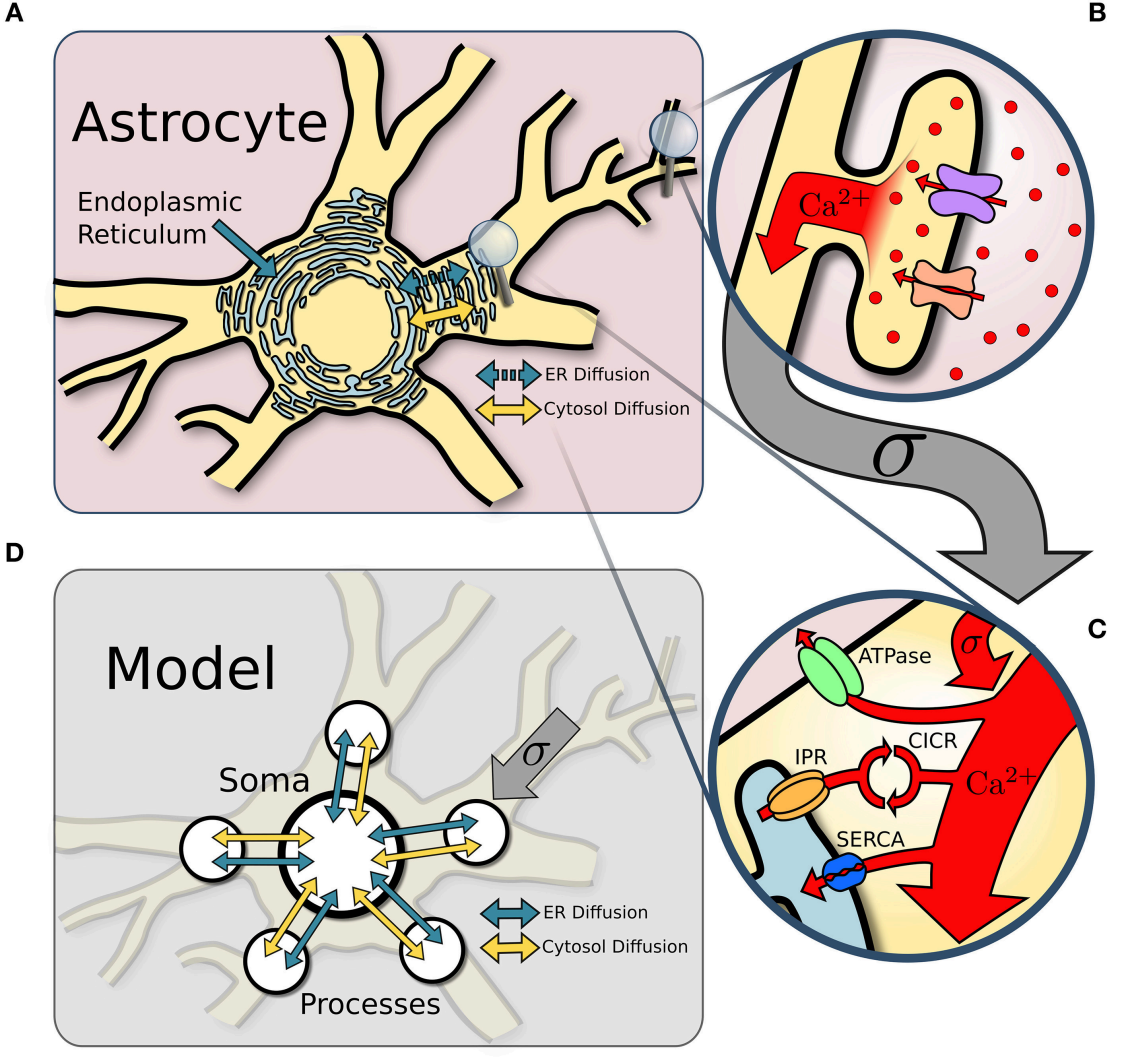
Model structure. **(A)** Overview of an astrocyte structure. The endoplasmic reticulum (ER) covers the astrocyte core (soma and major processes). **(B)** In response to neuronal activity, Ca^2+^ enters the perisynaptic astrocyte processes via the Na^+^/Ca^2+^ exchanger and other Ca^2+^ channels. **(C)** Ca^2+^ influx from perisynaptic processes sums at the larger ER-holding processes, which can activate Ca^2+^-induced-Ca^2+^-release (CICR). Removal of Ca^2+^ occurs via ATPase and SERCA pumps. **(D)** The astrocyte soma and five of its major processes are approximated with single point models connected via diffusion across both the ER and the cytosol.

We model the astrocyte as a soma with five major processes (see Figure 1). Each compartment defines its own single point model assuming homogeneization of Ca^2+^ concentration and interacts with the other compartments through diffusion. Compartmental Ca^2+^ in the cytosol and endoplasmic reticulum (ER) is expressed in terms of its concentrations $C_{I}^{\left(p_{i}\right)}$ and $C_{E}^{\left(p_{i}\right)}$ for process *i*, and $C_{I}^{\left(s\right)}$ and $C_{E}^{\left(s\right)}$ in the soma. Diffusion of Ca^2+^ between the soma and the process is proportional to the difference in Ca^2+^ concentration between the two compartments.

The Ca^2+^ dynamics is governed by a system of ODEs based on the non-dimensional functional model of Postnov et al. (2007). The explicit influence of the neurons is described by a source term that models the input of extracellular Ca^2+^ into the process cytosol via a variety of channels. Model dynamics are governed by the fluxes between cytosol and ER due to IPR activation and reuptake of Ca^2+^ by the ER through SERCA pumps. Finally, Ca^2+^ ions diffuse through the cytosol between soma and processes; we also include in the model the possibility for Ca^2+^ ions to diffuse through the ER. The Ca^2+^ thus satisfies the following set of equations:

(1) $$\begin{array}{rc} \tau_{c}\frac{dC_{I}^{\left(p_{i}\right)}}{dt} & =r+\sigma \xi_{i}+b_{4}\left(J_{r}-J_{p}\right)-C_{I}^{\left(p_{i}\right)}+J_{I,d}^{\left(p_{i}\right)} \end{array}$$

(2) $$\begin{array}{rc} \epsilon_{c}\tau_{c}\frac{dC_{E}^{\left(p_{i}\right)}}{dt} & =-J_{r}+J_{p}+J_{E,d}^{\left(p_{i}\right)} \end{array}$$

(3) $$\begin{array}{rc} \tau_{c}\frac{dC_{I}^{\left(s\right)}}{dt} & =r+b_{4}\left(J_{r}-J_{p}\right)-C_{I}^{\left(s\right)}-\overset{k}{\underset{i=1}{\sum }}J_{I,d}^{\left(p_{i}\right)} \end{array}$$

(4) $$\begin{array}{rc} \epsilon_{c}\tau_{c}\frac{dC_{E}^{\left(s\right)}}{dt} & =-J_{r}+J_{p}-\overset{k}{\underset{i=1}{\sum }}J_{E,d}^{\left(p_{i}\right)} \end{array}$$

where Equations 1 and 3 represent process Ca^2+^ dynamics and Equations 4 and 5 represent Ca^2+^ dynamics in the soma. Diffusion of Ca^2+^ ions between process *i* and the soma in the cytosol and the ER are respectively denoted by $J_{I,d}^{\left(p_{i}\right)}=D_{I}\left(C_{I}^{\left(s\right)}-C_{I}^{\left(p_{i}\right)}\right)$ and $J_{E,d}^{\left(p_{i}\right)}=D_{E}\left(C_{E}^{\left(s\right)}-C_{E}^{\left(p_{i}\right)}\right)$. The small parameter ϵ_*c*_ in Equation 5 controls the separation of timescales of the calcium in the cytosol and the ER.

Table 1 lists the model parameters and their default values.

**Table 1:** Default model parameters.

*b*_1_	Maximal rate of uptake from the cytosol to the ER	0.13
*b*_2_	Half-activation Ca^2+^ value of the IP_3_ channel	1
*b*_3_	Rate of non-active Ca^2+^ leak out of the ER	0.004
*b*_4_	Ratio of ER volume to cytosolic volume	50
ϵ_*c*_	Timescale separation between Ca^2+^ pools	0.04
τ_*c*_	Time constant for Ca^2+^ variations	8
*D*_*E*_	Diffusion coefficient for ER Ca^2+^	0
*D*_*I*_	Diffusion coefficient for cytosolic Ca^2+^	0.05
*r*	Baseline Ca^2+^ influx into process cytosol	0.31
*V*_*r*_	Ratio of soma to process volume	1.0

The parameter *r* controls the intrinsic Ca^2+^ excitability of each compartment, which may include a contribution from TRPA1 channels to basal Ca^2+^(Shigetomi et al., 2012). We set *r* = 0.31, which is just below the threshold for spontaneous Ca^2+^ spikes, as in Postnov et al. (2007). The effect of distal neuronal sources on the dynamics of Ca^2+^ in process *i* is captured by the source term σξ_*i*_, where ξ_*i*_ is sampled from *N*(0, 1). This fluctuating Ca^2+^ term is due in part to the Na^+^/Ca^2+^ exchanger (NCX), which is co-localized with Na^+^-dependent glutamate transporters, as well as neurotransmitter-gated Ca^2+^-permeable ion channels such as AMPA, NMDA, or P2X receptors (Kirischuk et al., 1997, 2007; Bazargani and Attwell, 2016). These Ca^2+^ signals combine with many others upon reaching the primary branch (Araque et al., 2014; Bindocci et al., 2017).

The diffusion coefficient *D*_*I*_ is set to 0.05 unless otherwise noted. The role played by the distribution and the geometry of the ER in glial cells has been under debate with evidence supporting varying degrees of ER interaction between domains of the astrocyte (Blaustein and Golovina, 2001; Petersen et al., 2001; Levine and Rabouille, 2005). To be consistent with these recent findings, we start by setting *D*_*E*_ = 0, but later consider the influence of non-zero values.

In Equations 1 and 5, the Ca^2+^ flux from ER to cytosol through IPR is represented by *J*_*r*_, while the flux from cytosol to ER due to SERCA pumps is represented by *J*_*p*_. To simplify the notation, we express these fluxes below in terms of Hill functions, defined by:

(5) $$\begin{array}{r} {\text{H}}_{n}\left(x,k\right)=\frac{x^{n}}{x^{n}+k^{n}} \end{array}$$

Excitability of Ca^2+^ dynamics is the result of Ca^2+^ flow between the cytosol and IP_3_-sensitive internal Ca^2+^ stores, which is initiated by the influx of either IP_3_ or extracellular Ca^2+^. There is a large spectrum of IPR models that vary in their consideration of IP_3_ and Ca^2+^. The primary characteristics that all IPR models share is that the steady-state open probability of the IPR channels are represented by a bell shaped function of Ca^2+^ (Dupont et al., 2016), which results in Ca^2+^-induced Ca^2+^ release (CICR) from internal stores. This work utilizes a representation of IPR flux taken from (Goldbeter et al., 1990) that assumes a sufficient level of IP_3_ and models the release of Ca^2+^ from the ER by an interaction between the two Ca^2+^ pools,

(6) $$\begin{array}{r} J_{r}\left(C_{E},C_{I}\right)={\text{H}}_{2}\left(C_{E},1\right){\text{H}}_{4}\left(C_{I},b_{2}\right) \end{array}$$

where *C*_*I*_ and *C*_*E*_ are the non-dimensional Ca^2+^ concentrations for the cytosol and ER respectively, and *b*_2_ sets the half-activation value of *C*_*I*_ for the IP_3_ channel. This model approximates IPR dynamics with a fast activation and slow inactivation of the channel by Ca^2+^ Dupont et al. (2016). We omit IP_3_ dynamics and diffusion in order to present the simplest possible framework through which a hierarchy of Ca^2+^ activity can be represented and explored.

Cells continuously pump Ca^2+^ from the cytosol into the ER through SERCA pumps on the ER membrane, modeled as

(7) $$\begin{array}{r} J_{p}\left(C_{I}\right)=b_{1}{\text{H}}_{2}\left(C_{I},1\right) \end{array}$$

where *b*_1_ is the maximum velocity of the reaction Dupont et al. (2016). SERCA pumps are influential in sustaining non-linear dynamics because they replenish the ER which is the source of excitable behavior.

### 2.2. Numerical method

The governing Equations (1-4) are solved numerically using a first order Euler-Maruyama method (Kloeden and Platen, 1992) to properly treat the stochastic neuronal input term σξ_*i*_. To establish the appropriate time discretization, several simulations were run with σ = 0, with default model parameters found in Table 1, except for *r*, which was set to *r* = 0.32, just beyond the threshold for spontaneous spike generation. To establish the appropriate step size we ran a simulation of 10,000 time units with successively smaller Δ*t*. We found that below Δ*t* = 0.5, the change in all spike times in the processes and in the soma satisfied

(8) $$\begin{array}{r} t_{spike_{\Delta t}}-t_{spike_{\frac{\Delta t}{2}}}\leq \Delta t \end{array}$$

where *t*_*spik*_*e*__Δ*t*__ is a spike time for a step size Δ*t*. Spike times are identified by *C*_*I*_ crossing a threshold of 1.2 while increasing Meyer and Stryer (1991). The numerical code is freely available and can be downloaded from https://github.com/FSUcilab/Compartmental_model_astrocytes.git

### 2.3. Correlated neural inputs

Astrocyte processes likely receive correlated input since the neuronal populations that contribute input to each process may overlap or have correlated activities (Averbeck et al., 2006; López-Hidalgo and Schummers, 2014). To allow flexible experimentation with correlated input, we construct a function that generates *k* input signals that have a fixed pairwise correlation coefficient ρ, where *k* is the number of active astrocyte processes. Starting with *k* uncorrelated signals **ξ** sampled from a normal distribution *N*(0, 1), we first compute the correlation matrix Σ such that Σ_*ij*_ = (1−δ_*ij*_)ρ+δ_*ij*_ (where δ_*ij*_ is the Kronecker Delta) and perform a Cholesky factorization Σ = *LL*^*T*^ (Johnson, 1994). Multiplication of *L* by the vector of uncorrelated signals **ξ** produces *k* signals with the desired correlation ρ:

(9) $$\begin{array}{r} \xi =L\tilde{\xi } \end{array}$$

### 2.4. Spike-triggered average

To analyze the relationship between the activity in the different compartments, we use spike-triggered averaging (STA) (Schwartz et al., 2006). For each soma spike, the Ca^2+^ trace of a process is selected within a time window preceding the soma spike. This ensemble of Ca^2+^ traces are then averaged. For comparison, we compute a baseline for which Ca^2+^ traces of the same length are selected at random times and averaged.

## 3. Results

### 3.1. Response of a compartmental astrocyte model to neuronal activity

Several studies have demonstrated that Ca^2+^ undergoes small, localized, oscillatory Ca^2+^ responses within the processes (Perea and Araque, 2002; Di Castro et al., 2011; Panatier et al., 2011; Araque et al., 2014; Bindocci et al., 2017). Integration of this activity may lead to local spiking in individual processes, which can recruit the soma and produce global spikes encompassing the soma and additional processes (Araque et al., 2014; Bindocci et al., 2017). In our model, each process *i* receives a neuronal input modeled as a Ca^2+^ source term ξ_*i*_(*t*). We explore how this leads to CICR-driven spikes in processes and soma. In general, nearby neurons can be correlated (Averbeck et al., 2006), and thus, the Ca^2+^ dynamics in the different processes might be correlated as well. As mentioned in section 2.3, we assume a pairwise correlation between input sources of ρ and explore the effect of ρ on the spiking frequency in the processes and the soma. We begin our simulations with zero signal correlation.

**Figure 2:**
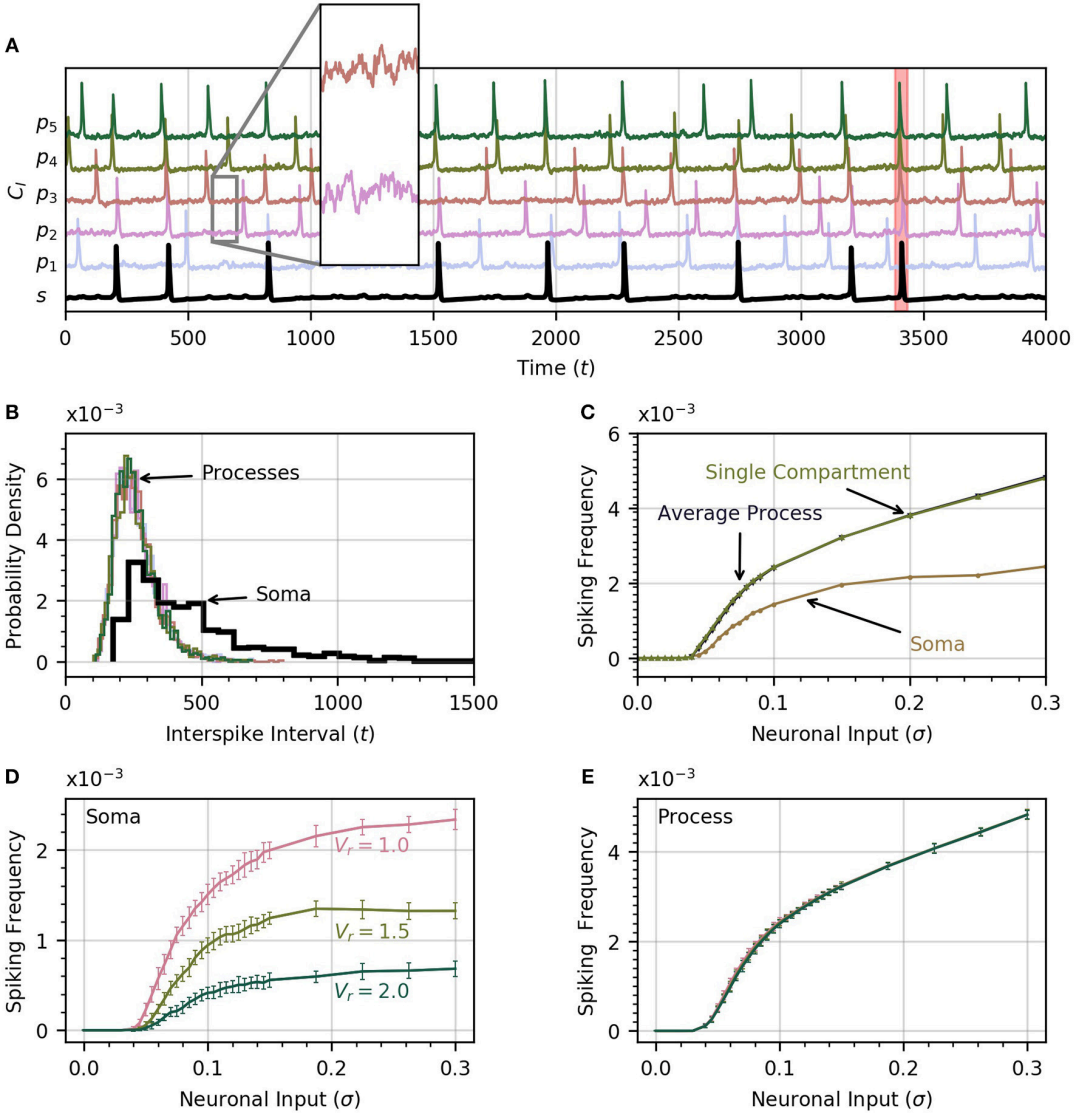
Astrocyte activity under random neuronal input. **(A)** Time course of cytosolic Ca^2+^ concentration in five processes (color) and the soma (black, bottom trace) over a 4000 time unit interval. All time traces have been normalized to their maximum value. Subthreshold oscillations are depicted in the inset. The shaded rectangle in the proximity of *t* = 3500 indicates a global event when at least four of five processes spike within 12.5 time units of the soma spike. **(B)** Histograms of interspike intervals for all processes and the soma are unimodal and follow an approximate log-normal distribution. **(C)** Both soma and average process spike frequency increase monotonically with neuronal input intensity. The variation of average process frequency with σ is identical to that for a single compartment model. **(D)** Spike frequency in the soma depends on the ratio *V*_*r*_ of soma to process volume. As *V*_*r*_ increases, the soma spikes less frequently. **(E)** On the other hand, spiking rates in the processes depend only very weakly on *V*_*r*_. When process and soma have equal volume, the process spikes at about half the rate as the soma. In (D,E), error bars were obtained by breaking up a simulation over 10^6^ time units into 20 equal intervals and computing the standard deviation across each interval. The same approach is adoped in subsequent experiments. Note that in our functional model, all units in this and subsequent figures are arbitrary. Simulation parameters: ρ = 0.0, *D*_*I*_ = 0.05 and *D*_*E*_ = 0 **(A–D)**, *V*_*r*_ = 1 **(A–C)**.

A time course of cytosolic Ca^2+^ in each process and in the soma is shown in Figure 2A, which includes an insert that zooms in on the subthreshold Ca^2+^ activity in two processes in response to the noisy neural input. The neuronal input signal amplitude (defined as the standard deviation of the signal) is initially set to σ = 0.2 . One of the soma spikes is identified as a global event, defined when at least four of five processes spike within a period of 12.5 time units from the soma spike. Thus our model covers the three scales of Ca^2+^ activity observed experimentally (Araque et al., 2014): a small scale for subthreshold Ca^2+^ activity due to neuronal inputs (hundreds of ms, spatially restricted to < 5μm), an intermediate timescale set by the process spikes (seconds, spatially extending over the length of a process), and the large scale of global events (up to 10 s, extending to most of the astrocyte core). The interval between successive spikes in a given process follows a sharp, approximately log-normal distribution (Figure 2B). The soma spikes at a lower frequency than the processes, as observed experimentally (Nimmerjahn et al., 2009; Kanemaru et al., 2014; Bindocci et al., 2017) and the distribution of interspike intervals is much wider for the soma than for the processes (Figure 2B), that is, there is more randomness in spike timing in the soma than in the processes.

As the intensity of the neuronal input σ is increased from 0 to 0.3, both the processes and the soma start to spike and the spiking rate increases monotonically with σ (Figure 2C). The soma spikes at a lower rate, and appears to saturate for σ>0.2. Below a threshold value of σ = 0.04, there are no process or soma spikes. The threshold is slightly higher for soma spikes that depend on one or more processes spiking before a sufficient amount of Ca^2+^ can diffuse into the soma. We examine the conditions that induce soma spikes in a later section. To determine how Ca^2+^ dynamics in the processes are affected by their connection to the soma, and via the soma by the other processes, we plot the spiking rate as a function of σ in a single compartment model. This curve is almost identical to the average spiking rate across processes for a low value of *D*_*I*_. This suggests that in this diffusion regime, the processes of our astrocyte model behave similarly to single compartment models, with little effect from interactions between compartments.

#### 3.1.1. Effect of larger soma volume

For simplicity, we have assumed that all compartments have equal surface and equal volume. However, a recent investigation into the three-dimensional morphology of astrocytes implied that the volume of the soma is about 1.5 times the volume of a major process (Bindocci et al., 2017). To consider the effect of a larger soma volume on the spiking activity, we define the soma to process volume ratio

(10) $$\begin{array}{r} V_{r}=\frac{V_{soma}}{V_{process}} \end{array}$$

under the assumption that all the processes have identical geometry.

Taking this volume ratio into account, the equations for Ca^2+^ dynamics in the soma become

(11) $$\begin{array}{rc} \tau_{c}V_{r}\frac{dC_{I}^{\left(s\right)}}{dt} & =V_{surf}\left(r+b_{4}\left(J_{r}-J_{p}-C_{I}^{\left(s\right)}\right)\right)-\overset{k}{\underset{i=1}{\sum }}J_{I,d}^{\left(p_{i}\right)} \end{array}$$

(12) $$\begin{array}{rc} \epsilon_{c}\tau_{c}V_{r}\frac{dC_{E}^{\left(s\right)}}{dt} & =V_{surf}\left(-J_{r}+J_{p}\right)-\overset{n}{\underset{i=1}{\sum }}J_{E,d}^{\left(p_{i}\right)} \end{array}$$

where $V_{surf}=V_{r}^{2/3}$ accounts for the increase in membrane surface area through which Ca^2+^ flux occurs. Thus, the increased volume in the soma slows down its Ca^2+^ dynamics.

Through Figures 2D,E, we examine the effect of volume ratio on spiking rate in the soma and the processes for the three volume ratios 1, 1.5, and 2. Larger volume ratios decrease the average soma spiking frequency for all values of neuronal input. On the other hand, the spiking rate in the processes are independent of the volume ratio. A larger soma volume requires a larger amount of Ca^2+^ to diffuse to the soma before $C_{I}^{\left(s\right)}$ rises enough to trigger CICR. In the remainder of the paper we set *V*_*r*_ = 1 to reduce the computation time, and allow the collection of a large enough number of soma spikes to perform statistical analysis.

### 3.2. Effects of input distribution onto the astrocyte model

#### 3.2.1. Correlated inputs trigger soma spikes more effectively and produce global events

**Figure 3:**
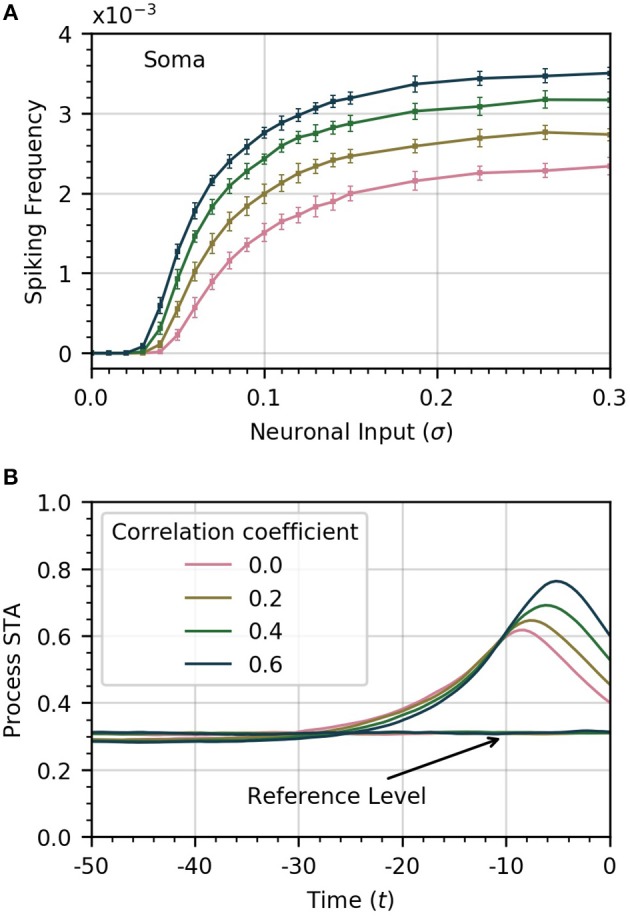
Increased correlation between neuronal inputs facilitates soma spike activity. **(A)** Variations of soma spike frequency with σ for different values of ρ. Higher input signal correlation raises the soma spiking frequency and decreases the threshold σ for soma spikes. **(B)** Spike-triggered average of *C*_*I*_ conditioned on the soma spikes within a window of 50 time units ahead of the soma spikes. At higher values of correlation, the process spikes are more concentrated and trigger closer to the soma spike. For reference, we show the average of *C*_*I*_ over random time windows. Simulation parameters: σ = 0.2, *D*_*I*_ = 0.05, *D*_*E*_ = 0.0, *V*_*r*_ = 1.0.

The amplitude of inputs is not the only factor that affects astrocyte Ca^2+^ dynamics. Since neurons in a given cortical area can have correlated activities, it is likely the case that the inputs to the different astrocyte processes also exhibit a (potentially variable) degree of correlation (Averbeck et al., 2006). To simulate this correlation, we generate input signals ξ_*i*_(*t*) with pairwise correlation ρ. We then compute the activity in the processes and soma as a function of σ for different values of ρ. Frequency curves are shown in Figure 3A. As the correlation coefficient increases, soma spiking frequency increases, and plateaus at higher values.

We also find that higher correlation between Ca^2+^ influx signals decreases the threshold σ necessary for the soma to spike. In Figure 3B, we plot the STA of process activity conditioned on soma spikes for various values of ρ. At higher levels of correlation, the STA is sharper and closer to the soma spike. This suggests that synchrony between process spikes facilitates soma spiking.

**Figure 4:**
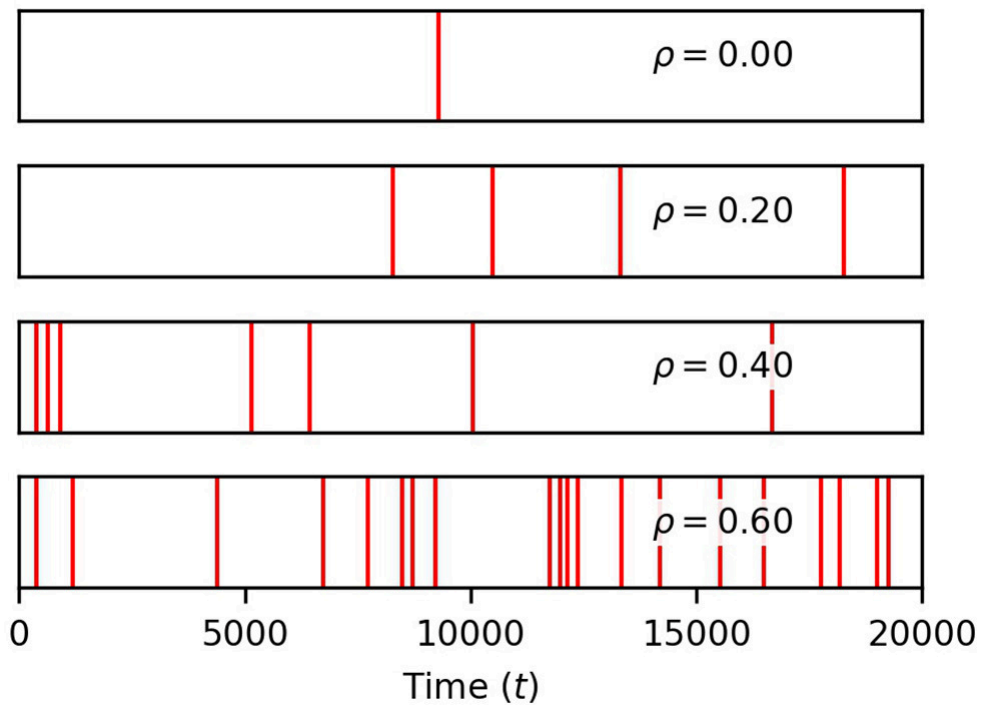
Correlation between neuronal inputs induce global events. Each red vertical bar marks the occurrence of a global event (four or more process spikes within a window of 12.5 time units of a soma spike). Their density rises with ρ. Simulation parameters: σ = 0.2, *D*_*I*_ = 0.05, *D*_*E*_ = 0.0, *V*_*r*_ = 1.0 .

Finally, we show in Figure 4 that the occurrence of global events strongly increases with ρ. Thus, while finite correlation between input sources have a moderate effect on soma spiking, they greatly increase the number of global events. For the remainder of the paper, we set the pairwise correlation coefficient between neuronal signals to ρ = 0.2, unless explicitly stated otherwise.

#### 3.2.2. Concentrating neuronal inputs helps trigger soma spikes

Up until this point, we have assumed a uniform distribution of Ca^2+^ due to neuronal activity. However, our model provides the ability to stagger the level of Ca^2+^ input between processes. We wish to better understand whether neuronal inputs should be distributed over many processes to effectively evoke somatic Ca^2+^ spikes.

**Figure 5:**
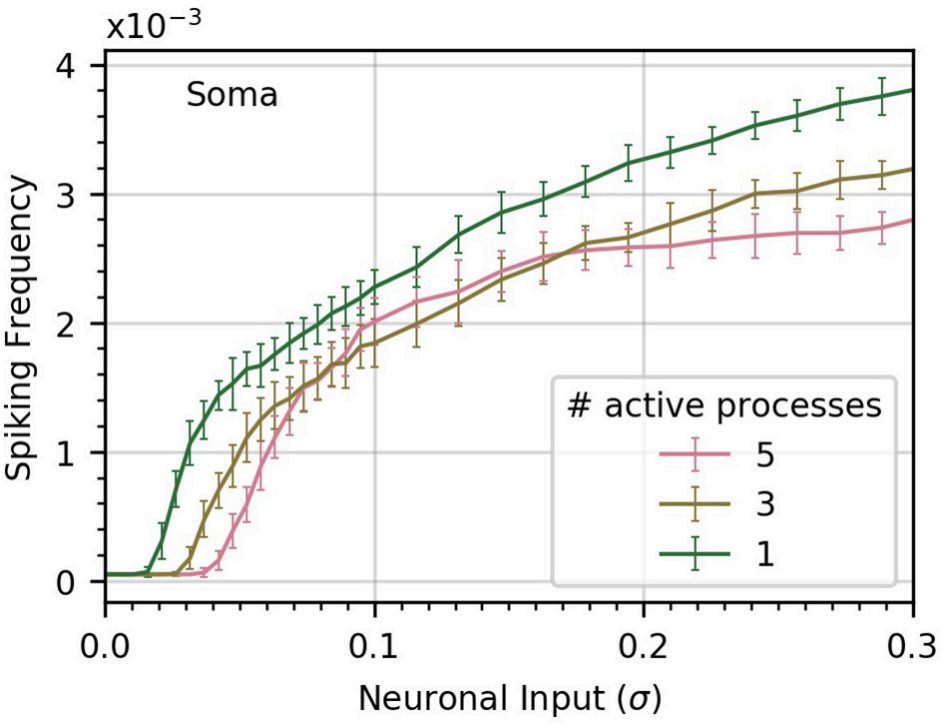
Concentrating inputs into fewer processes facilitates soma spiking. Curves (a-c) correspond respectively to 5, 3, and 1 processes receiving neuronal input; these are the active processes. By definition, σ is the standard deviation of the neuronal input signal into five processes, as in previous figures. As the number of active processes is decreased, σ is scaled up to maintain a constant standard deviation of the sum of the inputs. Concentrating the neuronal inputs into fewer processes increases soma spike rate across most of the σ range and decreases the threshold σ for soma spiking. Simulation parameters: ρ = 0.2, *D*_*I*_ = 0.05, *D*_*E*_ = 0.0 .

To gain some insight into this question, we perform three experiments in which we vary the number of processes subject to neuronal input (Figure 5). In the base case, all five processes receive the same level of neuronal activity, denoted by σ. In the second case, each of three processes receives a level σ_3_, and in the third case, a single process is active, with neuronal activity σ_1_. We choose the levels σ_1_ and σ_3_ to ensure that in each case the sum of all active signals has identical variance.

More generally, consider the sum *S*_*m*_ of *m* signals *s*_*i*_ with pairwise correlation ρ and variance σ^2^. It can easily be shown that the standard deviation of *S*_*m*_ is given by

(13) $$\begin{array}{r} \sigma \left(S_{m}\right)=\sigma \sqrt{m+m\left(m-1\right)\rho }. \end{array}$$

With the help of Equation 14, we obtain relationships that ensure comparative total input between the standard deviations of the process neuronal input when one and three processes are active:

(14) $$\begin{array}{rc} \sigma_{1} & =\sigma \sqrt{5+20\rho }, \end{array}$$

(15) $$\begin{array}{rc} \sigma_{3} & =\sigma \frac{\sqrt{5+20\rho }}{\sqrt{3+6\rho }}. \end{array}$$

We find that concentrating inputs onto fewer processes slightly increases spiking frequency in the soma, and decreases the threshold σ required to evoke soma spiking (Figure 5). At higher values of σ, concentrating inputs in fewer processes only has a small effect on somatic frequency. This suggests that neuronal inputs concentrated in only a few processes are more likely to trigger soma spikes than if they are distributed throughout the astrocyte processes.

### 3.3. Compartmental diffusion effects

Calcium gradients across neighboring cell regions are accounted for in our model by diffusion fluxes between adjacent processes, either through the cytosol (*J*_*I, d*_) or through the ER (*J*_*E, d*_). Accordingly, we set out to investigate how variations of diffusion coefficients *D*_*I*_ and *D*_*E*_ affect somatic spiking.

#### 3.3.1. Diffusion between soma and process cytosol

**Figure 6:**
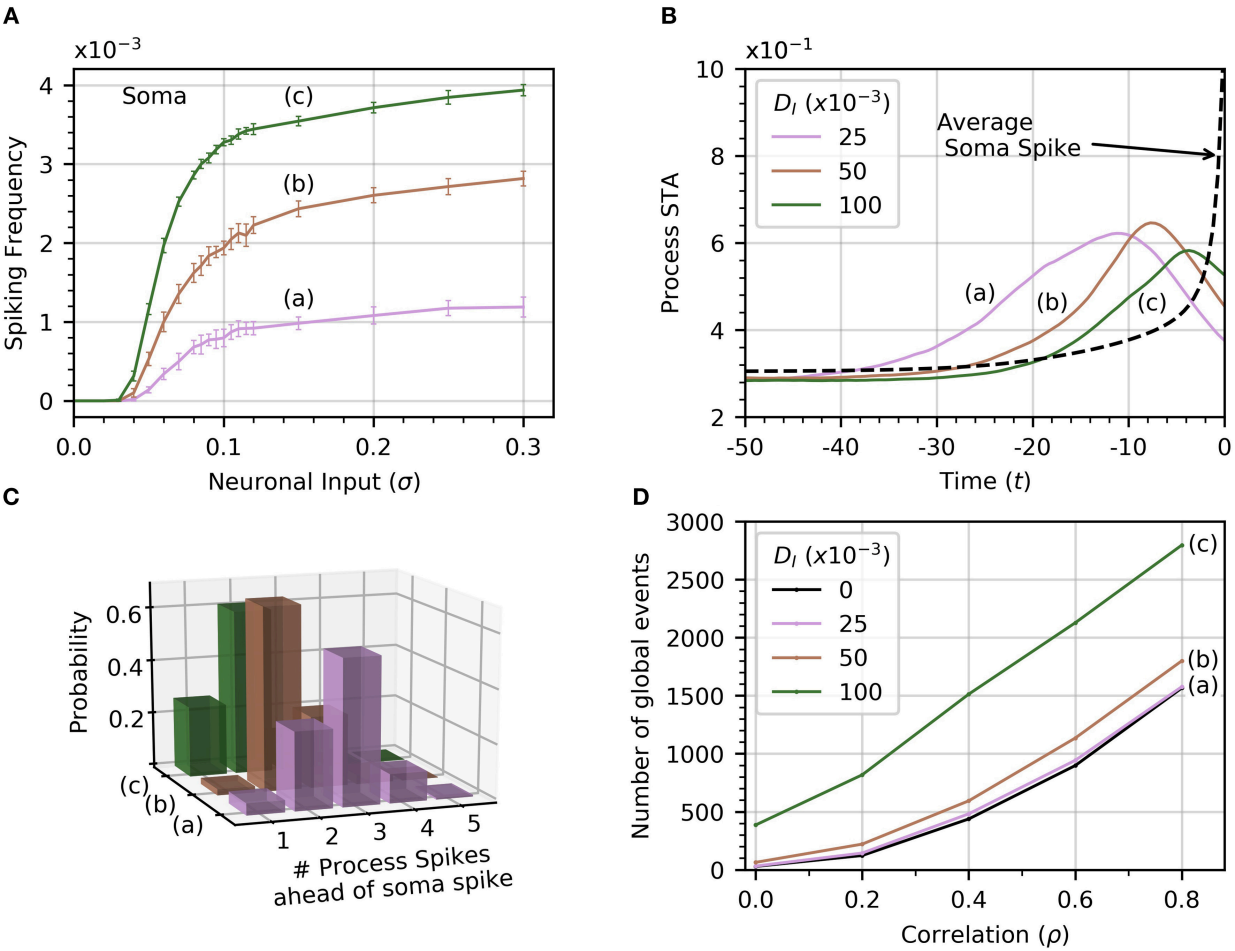
Effect of cytosolic diffusion on soma and global activity. **(A)** Soma spiking rate increases with cytosolic diffusion *D*_*I*_ for all levels of neuronal input. Higher *D*_*I*_ also decreases the threshold input for soma spikes. In **(A,B,D)**, curves (a) through (c) correspond to *D*_*I*_ = 0.025, 0.05, 0.1, respectively. **(B)** Spike-triggered averages of $C_{I}^{\left(p\right)}$ in a single process (all processes produce a very similar curve) were computed in a window of 50 times units ahead of the soma spikes. Higher *D*_*I*_ shortens the average interval between soma spike and preceding process spikes. **(C)** The distribution of number of process spikes preceding a soma spike shifts toward lower values at higher *D*_*I*_. The number of process spikes were only counted within a window of 30 time units ahead of the soma spikes. **(D)** Variations of the number of global events (defined by at least four processes spiking within a time window of 25, with or without a soma spike) with ρ. Cytosolic diffusion determines whether the soma directly influences the number of global events, or whether they are due to random process spike co-occurrences. As input correlation increases in the absence of soma activity (*D*_*I*_ = 0), so do the number of randomly occurring global events (dashed curve). Note that in the absence of cytosolic diffusion, the soma does not spike. When *D*_*I*_ increases to 0.025, the soma begins to spike [panel **A**, curve (a)], but the number of global events does not increase significantly. Further increases of *D*_*I*_ to 0.05 and 0.1 shows a substantial increase in the number of global events, which are almost certainly associated with soma spikes. That this is so is substantiated by noticing that process spiking frequency is unaffected by *D*_*I*_, as seen in Figure 2C, which shows that the process spiking frequency at *D*_*I*_ = 0.05 is the same as in the single compartment (equivalent *D*_*I*_ = 0). Given a sufficiently strong cytosolic diffusion (*D*_*I*_ = 0.1), soma spikes help generate significantly more global events than those generated by input signal correlation alone. Simulation parameters: *D*_*E*_ = 0, σ = 0.2, *V*_*r*_ = 1 .

The soma receives no input from neuronal activity, so it only becomes active because of the diffusion of cytosolic Ca^2+^ from spiking processes. Thus, the soma spiking rate should increase with *D*_*I*_, as seen in Figure 6A. Curves (a) through (c) correspond to increasing levels of *D*_*I*_ (0.025, 0.050, 0.1). Increasing *D*_*I*_ also slightly decreases the threshold value of σ for soma spikes. To determine how the rate of diffusive Ca^2+^ transfer impacts the relationship between process and soma spiking, we plot the average spike-triggered average of processes Ca^2+^ conditioned on soma spikes for different values of *D*_*I*_ in Figure 6B. At higher values of *D*_*I*_, the STA of process activity narrows and moves closer to the soma spike, suggesting that for higher values of *D*_*I*_ fewer process spikes occurring within a short time frame are needed to elicit a soma spike. To confirm this, we plot the likelihood of different process spike counts preceding a soma spike for several values of cytosolic diffusion. This information is summarized in Figure 6C. It shows that for *D*_*I*_ = 0.05 or less, most soma spikes are triggered only once two or three process spikes occur close to each other. Thus, soma spikes occur less frequently and less regularly than process spikes, explaining the wider distribution of ISI for soma spikes (Figure 2B).

#### 3.3.2. Are global events random?

From Figure 4, we recall that increasing ρ increases the number of global events. Are these events the result of spike propagation from the soma to other processes, or are they simply random synchronizations between process spikes due to the correlation between neuronal inputs? To answer this question, we set *D*_*I*_ = 0 to ensure that events that involve four or more process spikes within 25 time units can only occur as random coincidence of process spikes. We plot the number of such global events for increasing signal correlation in Figure 6D. When *D*_*I*_ lies between 0.025 and 0.05, there is little difference with the number of global events found for *D*_*I*_ = 0. Thus, these global events are simply due to random occurrences. However, when *D*_*I*_ = 0.1, the number of global events rises markedly. This demonstrates that the soma is involved with the global event generation. Therefore, the strength of diffusion between compartments can change the nature of global events.

#### 3.3.3. Diffusion within the endoplasmic reticulum

**Figure 7:**
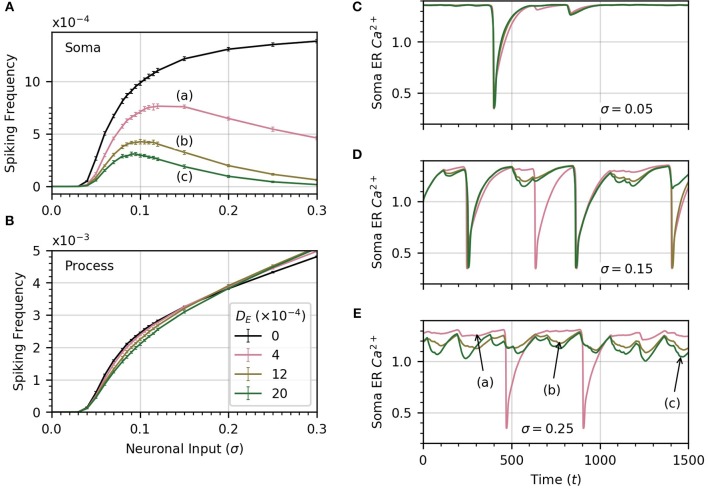
ER diffusion leads to non-monotonic variation of soma spike frequency with σ. **(A)** Non-monotonic variations of soma spike frequency with σ for non-zero *D*_*E*_. The maximum soma spike frequency decreases and shifts toward lower σ for higher values of *D*_*E*_ (from a to c). The *D*_*E*_ = 0 curve is drawn in black. **(B)** The process spike frequency is mostly independent of *D*_*E*_, except in the region near σ = 0.1 where the frequency is slightly elevated at lower *D*_*E*_. Note that the error bars have similar magnitude to those in **(A)**. Simulation parameters: ρ = 0.2, *D*_*I*_ = 0.05, *V*_*r*_ = 1 . **(C–E)** The bell-shaped curves in **(A)** are explained by examining the time course of soma ER Ca^2+^ for different levels of *D*_*E*_ at three levels of σ. At low σ **(C)**, the level of the soma ER Ca^2+^ does not vary significantly with *D*_*E*_. **(D,E)** At high σ, $C_{E}^{\left(s\right)}$ depletion increases with *D*_*E*_, leading to missed soma spikes. Large downward deflections result from soma spikes while small downward deflections result from process spikes. All simulations were conducted with the same initial random seed to generate a consistent sequence of process spikes as *D*_*E*_ is varied. Simulation parameters: *D*_*I*_ = 0.05, ρ = 0.2 .

Up until now, we have assumed that there was no connection between process and soma ER. We now relax this assumption and consider the impact of diffusion between the two. Since the extent of diffusion within the astrocyte ER is unknown, we consider a range of ER diffusivities. We set *D*_*I*_ = 0.05, which allows high frequency spiking in the soma when no diffusion occurs between ERs (see Figure 6A). Next, we compute the average soma frequency as a function of σ for three values of *D*_*E*_ in the range 0 to 0.002 . As soon as *D*_*E*_ > 0, the relationship between soma frequency and σ becomes non-monotonic (Figure 7A). As σ increases, the spike frequency in the soma initially increases toward a maximum value, and then decreases as σ continues to increase. This effect becomes stronger, limiting the maximum soma spike frequency, for larger values of *D*_*E*_. Thus, diffusion between the soma and process ER impedes soma activity at high levels of neuronal inputs. Spiking activity in each process remains unchanged as shown in Figure 7B.

To explain why the soma spikes at a lower rate at high σ, we note that two opposite factors shape the relationship between the rate of spikes in the soma and the neuronal input intensity. As σ increases initially, the spike rate in the processes rises, which leads to more spikes in the soma. Accordingly, the first part of the curves in Figure 7A shows an increase in spiking frequency with σ. However, this picture changes at higher σ when *D*_*E*_ > 0. In that case, each process spike causes a flow of Ca^2+^ from the soma ER to the process ER, because process ER Ca^2+^ has rushed into the process cytosol. Thus, following a process spike, the soma ER becomes transiently depleted. The opposite is also true: a soma spike causes flow of Ca^2+^ from the process ER to the soma ER, but the soma spikes less frequently than the processes. Therefore, the net effect is that soma ER Ca^2+^ level will decrease, this effect becoming stronger with larger σ. This lowers soma excitability, lowering the soma spike frequency. (Below, we illustrate the ER Ca^2+^ depletion by process spikes.)

To summarize, at low σ, the spike rate increases as the slope of frequency vs σ is quite large. On the other hand, as σ increases, the higher spike rate due to higher excitation slows down, while the soma ER gets further depleted with increased process spike frequency. Around σ = 0.1, this depletion of soma ER Ca^2+^ lowers soma excitability so much that soma spike rate decreases with σ.

We confirm the above by displaying the time course of soma ER Ca^2+^ for $D_{E}=\left(4,12,20\right)\times 10^{-4}$, at three values of σ in panels C (σ = 0.05), D (σ = 0.15), E (σ = 0.25), of Figure 7. At low σ, the time course of the soma ER Ca^2+^ does not vary significantly over the range of *D*_*E*_ considered. At higher values of σ (Figures 7D,E), the differences between ER soma Ca^2+^ at different *D*_*E*_ levels grow, which leads to some missed soma spikes. Large downward deflections result from soma spikes while small downward deflections result from process spikes. At higher values of σ, the soma ER can be depleted for *D*_*E*_ > 0, causing the soma to miss some spikes.

## 4. Discussion

Astrocytes play a range of roles on neural circuits at different spatial and temporal scales. This range of astrocytic effects on neural circuits is mediated by a hierarchy of Ca^2+^ events in astrocytes. In turn, these events are a response to neuronal activity (Shigetomi et al., 2016; Bindocci et al., 2017). These events range from fast, localized transients, to slower global events that recruit the soma and major processes. To better understand the spatiotemporal propagation of Ca^2+^ signals in the cell and how these signals might be altered in neurodegenerative diseases, we need to investigate how neuronal inputs give rise to this hierarchy of Ca^2+^ events.

In this paper, we applied a compartmental model to investigate how the properties of neuronal inputs give rise to a variety of Ca^2+^ events in astrocytes. The model is adapted from the single compartment, functional model of Postnov et al. (2007) that describes astrocyte response to perisynaptic Ca^2+^ influx. We added compartments representing major astrocyte processes that couple to the soma through diffusion.

In this simple model, we demonstrated that the Ca^2+^ influx into each process in response to neuronal activity controls the occurrence of process Ca^2+^ spikes, and in turn soma Ca^2+^ spikes. The degree of correlation between the process neuronal inputs determines the frequency of global events involving the soma and at least four processes. These global events, in the model, are simply due to the random co-occurrence of process spikes in response to correlated neuronal inputs, unless cytosolic diffusion is large enough for soma spikes to trigger spikes in processes. Our simulations also indicate that the exchange of Ca^2+^ between the soma and process ER compartments severely curtails the number of somatic spikes in response to high levels of neural activity.

### 4.1. A first model to build intuition

Our goal was to build an initial model that incorporates the spatial structure of astrocytes to explore their hierarchy of Ca^2+^ dynamics. This model investigates questions related to astrocyte geometry, Ca^2+^ diffusion across its compartments, the distribution of neuronal inputs received by its perisynaptic processes and the effect of these inputs on Ca^2+^ activity in both the soma and major processes. The adoption of a simple model as a starting point in our investigation helps build intuition about the origin of astrocytic Ca^2+^ events. Indeed, only a small number of variables interact to produce these events. A more complex model that incorporates details of astrocyte geometry and signaling pathways would be required to match experimental data. Such models, however, come at the expense of a large number of parameters and variables, which would make the analysis less intuitive and prone to fine tuning of parameters. Our model is built on reasonable assumptions, can be extended to ask more detailed questions, and may guide further model development and experiments since it provides a series of testable predictions.

One important assumption in our work is that all major processes possess Ca^2+^ excitability, and that neuronal activity only affects processes, not the soma. Experiments have shown that neuronal activity causes fast, local Ca^2+^ transients in distal, fine process branches that do not contain IP_3_ receptors and therefore show no Ca^2+^ excitability. These Ca^2+^ events may be triggered by the opening of Ca^2+^-permeable channels in response to neuronal activity (Hamilton et al., 2008; Shigetomi et al., 2012; Lind et al., 2013; Srinivasan et al., 2015). Herein, we model the effects of the distant Ca^2+^ transients onto the major processes as a Gaussian distribution of Ca^2+^ input. We have implicitly assumed a constant level of IP_3_, such that integration of the random Ca^2+^ influx events could trigger CICR. This is because Ca^2+^ entry through channels can happen without activation of G-protein coupled receptors that lead to the production of IP_3_, and because IP_3_ concentration within the astrocyte should equilibrate faster than the Ca^2+^ concentration. Since Ca^2+^ excitability in the processes may not require IP_3_ receptors (Srinivasan et al., 2015), we did not use a model of Ca^2+^ excitability that explicitly depends on intracellular IP_3_ levels. There is evidence, however, that the Ca^2+^ levels resulting from Ca^2+^ spikes may affect IP_3_ levels, which may be important for the production of multiple modes of Ca^2+^ dynamics (De Pitta et al., 2009).

In addition, all processes have a basal rate of Ca^2+^ influx that brings them close to threshold for a Ca^2+^ spike in the absence of neuronal activity. We identify the level of astrocyte excitability with the spike frequency in the soma and in the processes. The implicit assumption is that spiking rate in response to sustained activity serves as a good measure of the effectiveness of the neuronal input patterns in evoking Ca^2+^ responses.

### 4.2. Control of astrocyte *Ca*^2+^ dynamics by neuronal activity

The framework underlying this work, and discussed further below, was built up from a series of experimental results summarized by Carmignoto (2000), Volterra and Meldolesi (2005), and Zorec et al. (2012), which in turn led to the presentation in Araque et al. (2014). According to this framework, neuronal activity causes fast, localized Ca^2+^ events that occur in distal branches, which can result in regenerative Ca^2+^ events in the main process through a process of integration not unlike integration of synaptic inputs by neurons. As neuronal activity increases, spikes in processes can in turn be integrated and trigger soma spikes and global events. To our knowledge, however, this has not been demonstrated experimentally and recent work, while not contradicting this simple framework, suggest that Ca^2+^ dynamics in astrocytes may exhibit a wide range of events (Wu et al., 2014; Bindocci et al., 2017), possibly with more complex rules than assumed here.

The model generates four types of Ca^2+^ events: (1) fast transients due to neuronal activity onto each process; (2) process spikes; (3) soma spikes; and (4) global events that involve spikes in soma and several processes within a short time window. Fast transients (1) are always produced as long as there is neuronal activity (σ > 0). Astrocyte processes integrate fast Ca^2+^ transients caused by neuronal inputs, leading to process spikes. The soma Ca^2+^ rises due to process spikes. We have not observed soma spikes (3) without process spikes (2) and rarely observed global events (4) without soma spikes (3). Thus the chain (1)-(4) of observable Ca^2+^ events defines a hierarchy, whereby an event high in the hierarchy only occurs when preceding events in the hierarchy have already been reached.

The particular Ca^2+^ events (1)-(4) observed in the hierarchy depend on the parameters σ and ρ, which respectively describe neuronal activity and input correlation. At very low σ, we do not observe spikes in either processes or soma: only fast Ca^2+^ transients (1) occur in the processes. At higher σ, processes begin to spike (2) and their spike rate increases with σ. The spiking frequency in the process reproduces the behavior of the spiking rate in a single compartment model with identical σ (Figure 2B). As σ is increased further, soma spikes appear and their spiking rate dependence on σ is similar to that of the processes. The soma spikes less frequently than the processes, as observed experimentally (Araque et al., 2014; Bindocci et al., 2017). Taking into account the fact that the soma volume is about 1.5 times the volume of a major process leads to an even lower soma spike rate than in the processes, since larger volume ratio slows down Ca^2+^ variations.

The role played by correlation between neuronal inputs on astrocyte Ca^2+^ dynamics differs from that played by σ. Increasing ρ has practically no effect on the average process spiking rate and only a modest effect on that of the soma (Figure 3A), except at low σ. In that case, increasing ρ facilitates soma spikes, and lowers the σ threshold for soma spikes. Beyond this effect of correlation on the soma spike rate, increasing ρ mainly increases the frequency of global events. That is, the proportion of soma spikes that are global events increases with ρ, but not with σ. Thus, the amount of neuronal input onto the processes controls the rate of spiking in the processes and in the soma, while the degree of correlation between neuronal inputs controls the frequency of global events.

### 4.3. Implications for the role of astrocytes in neural computations

In addition to many roles played by astrocytes as neuron support cells and regulators of blood flow, there is growing evidence that astrocytes also modulate the activity of neural circuits by acting on neuronal excitability and on synaptic activity and plasticity. It has been suggested that the spatiotemporal distribution of neuronal activity determines the profile of Ca^2+^ transients evoked in astrocytes. This in turn determines the extent of synaptic modifications by astrocytes (Araque et al., 2014). According to that hypothesis, localized and short-lived synaptic activity only evokes fast, local astrocyte transients–event (1) above–which in turn only affect synapses directly involved in triggering the fast Ca^2+^ events. Stronger, longer lasting, or slightly more widespread synaptic activity in the same area could trigger a spike in one major process (2). In turn, this affects synapses under its control, active or not. Finally, synaptic activity occurring in more than one process may evoke somatic spikes and global events, (3) and (4), depending on the degree of synchrony of synaptic activation. These events in turn may lead to synaptic plasticity in all synapses covered by the astrocyte. Araque's hypothesis only holds if a hierarchy of Ca^2+^ events results from a hierarchy of network synaptic activity. Our results support this hypothesis by demonstrating that the different types of astrocyte Ca^2+^ events follow such a hierarchy. The higher in the hierarchy, the more synapses over a larger area may be affected synchronously by astrocyte signaling.

Our results may also explain why astrocytes in the visual cortex maps have sharper tuning curves than do neurons. Schummers et al. (2008) measured global events in astrocytes in response to visual inputs with different orientation or spatial frequency. Astrocytes responded according to which orientation domain they belonged to, but their tuning curves were sharper than that of neurons within the same domain. These sharper tuning curves may result from the fact that the distributed activation of synapses over the whole astrocyte must exhibit a significant degree of temporal correlation to induce global events. However, by the same reasoning, astrocytes located around pinwheel centers, which do not receive tuned inputs, should therefore have an untuned response. This does not seem to be the case, as astrocytes located close to a pinwheel center still exhibit sharp tuning curves (López-Hidalgo and Schummers, 2014).

### 4.4. ER geometry affects the soma response to inputs

CICR generates spikes while somatic and global events rely on diffusion between processes and the soma. Therefore the ER, which is the main store of astrocytic Ca^2+^, plays an important role in these Ca^2+^ events. Spiking in the soma occurs as a response to increased levels of neuronal activity when the ERs in the soma and processes connect through diffusion. Setting *D*_*E*_ to a non-zero value resulted in a qualitative change in the relationship between the amount of neural activity and the soma spike frequency. More specifically, the graph of average frequency vs. σ becomes non-monotonic, initially increasing at low values of σ, then decreasing back toward 0. During a process spike ER, Ca^2+^ in the process becomes depleted, which leads to diffusion of Ca^2+^ from the soma ER to the ER in the processes. Since the processes spike more frequently than the soma, the net result is a decrease of Ca^2+^ in the soma ER and lower soma excitability.

We note that in this model of Ca^2+^ excitability, Ca^2+^ in ER plays the role of the recovery variable. Thus, the loss of Ca^2+^ by the soma ER engenders a decrease in soma excitability as σ increases from the lower levels. With a different model with de-inactivation of IP_3_R channels by Ca^2+^ as the recovery variable, we may not see the drop in soma spike frequency as some ER Ca^2+^ becomes depleted, but rather a drop in soma spike amplitude.

Our model does take into account the effect of store-operated Ca^2+^ channels. Store-operated Ca^2+^ entry would activate once the ER Ca^2+^ reaches a low level, potentially resulting in an influx of Ca^2+^ through the cytoplasm. This could prevent the decrease in soma excitability at high σ. Thus, a modification in the parametrization of Ca^2+^ excitability in the model might impact cause and effect between processes and soma ER.

### 4.5. Relevance to neurodegenerative diseases

Pathological changes in astrocytes alter Ca^2+^ activity in several forms of neurodegenerative disease (Nedergaard et al., 2010; Hamby et al., 2012; Sofroniew, 2014; Ben Haim et al., 2015; Rodŕıguez-Arellano et al., 2016; Verkhratsky et al., 2016). Neurological diseases can be viewed as a homeostatic failure and astrocytes are affected through alterations in multiple disease-specific homeostatic mechanisms generally known as reactive gliosis (Sofroniew, 2005; Nedergaard et al., 2010; Rodŕıguez-Arellano et al., 2016; Verkhratsky et al., 2016). Reactive gliosis is an evolutionarily conserved defense mechanism associated with neurodegenerative disease; the resultant remodeling undergone by astrocytes can contribute to pathological progression and alter Ca^2+^ dynamics (Verkhratsky et al., 2016).

In Alzheimer's disease (AD), β-amyloid buildup has been shown to initiate reactivity in astrocytes (Alberdi et al., 2013). For instance, exposure of astrocytes to β-amyloid in hippocampal slices resulted in Ca^2+^ release from the ER, which instigated astroglial reactivity (Alberdi et al., 2013). β-amyloid plaques also lead to aberrant physiology in the form of spontaneous Ca^2+^ oscillations as well as intercellular Ca^2+^ waves (Kuchibhotla et al., 2009; Lim et al., 2014).

Prior to the development of β-amyloid plaques, astrocytes undergo atrophy as a result of reactive gliosis (Verkhratsky et al., 2016). Atrophy in turn leads to decreased volume of the soma as well as a reduction in the number of primary processes (Olabarria et al., 2010; Yeh et al., 2011; Kulijewicz-Nawrot et al., 2012). A model like the one developed here may be used to predict the consequences of these morphological changes, together with functional changes in Ca^2+^ influx, on Ca^2+^ spiking and global events.

## 5. Conclusion

We have demonstrated that a functional compartmental model can answer questions about the rules that govern the generation of Ca^2+^ events observed in astrocytes. A parametric study conducted with respect to Ca^2+^ influx intensity and pairwise correlation, *D*_*I*_, and *D*_*E*_, suggests that the variability of neuronal activity seen by the astrocyte sets the spiking frequency in both the processes and in the soma. The correlation between the neuronal activity in the different processes controls the number of global events. Our calculations also suggest that diffusion between the ER of the soma and of the major processes has an important effect on the dynamics of Ca^2+^ events in astrocytes. A detailed look at the mechanics behind the global events and the conditions under which synchronization occurs remains a subject of future investigation.

## Author contributions

All authors listed have made a substantial, direct and intellectual contribution to the work, and approved it for publication.

### Conflict of interest statement

The authors declare that the research was conducted in the absence of any commercial or financial relationships that could be construed as a potential conflict of interest.
